# Polygenic risk scores in routine genetic diagnostics: what lies ahead?

**DOI:** 10.1007/s12687-025-00835-x

**Published:** 2025-11-20

**Authors:** Peter Lauffer, W. van Weelden, M.M. van Haelst, Philip R. Jansen

**Affiliations:** 1 Amsterdam UMC, Department of Human Genetics, University of Amsterdam, Amsterdam, Netherlands; 2 Amsterdam Reproduction and Development, Amsterdam, Netherlands; 3 Emma Center for Personalized Medicine, Amsterdam UMC, Amsterdam, The Netherlands; 4 Department of Complex Trait Genetics, Center for Neurogenomics and Cognitive Research, VU University, Amsterdam, Netherlands

**Keywords:** Polygenic risk scores, Clinical genetics, Genetic testing, Complex genetics, Rare disease genetics

## Abstract

Polygenic risk scores (PRS) have emerged as a potential tool for predicting complex genetic traits and disorders, which may complement traditional rare variant testing. As genome-wide association studies (GWAS) expand, PRS predictive accuracy improves, yet its role in clinical genetics remains undefined. Here, we discuss four scenarios for PRS integration into diagnostic workflows: (1) PRS as a first-tier screen to stratify patients for rare variant testing; (2) parallel testing with whole-genome sequencing (WGS) to capture both rare and common variant contributions; (3) selection between PRS and rare variant testing guided by clinical characteristics; and (4) PRS application in rare variant-negative cases to identify likely polygenic etiologies. We highlight different trade-offs of each approach, which include costs, turnaround time, diagnostic efficiency, and risk of secondary findings. While PRS shows promise in conditions with both monogenic and polygenic contributions, challenges remain in defining risk thresholds, equal accuracy across (non-European) ancestries, and integrating PRS into clinical decision-making. Although not yet standard practice, we envision PRS is likely to play an increasing role in genetic diagnostics, necessitating collaboration between clinicians and laboratory geneticists to optimize its application.

## Introduction

Polygenic risk scores (PRS) have gained momentum as a way to understand, and eventually predict, complex genetic traits and disorders (Lewis and Vassos 2020). With ongoing increases in number and sample sizes of genome-wide association studies (GWAS), reaching over 1 million participants (Jansen et al. 2019), there has been a rise in predictive accuracy of PRS based on the statistical associations of many common genetic risk variants. As clinicians often face challenges in deciding when a set of symptoms is due to a monogenic cause versus a likely polygenic etiology, these improved PRSs may eventually complement existing genetic tests by providing quantitative risk information for certain disorders in daily practice. However, clinical geneticists and counselors still need to define the optimal position of PRS in the current diagnostic trajectory, and laboratories need to develop workflows in which PRS can be analyzed and interpreted. A discussion on how to integrate the analysis of complex genetic factors (often obtained by SNP array genotyping) with more traditional (rare variant) sequencing methods in the same diagnostic trajectory is currently lacking, and is of importance to anticipate ongoing developments in the field. To this end, we here provide several scenarios of how PRS may be implemented in daily clinical practice.

## PRS as a first-tier test

**Fig. 1:**
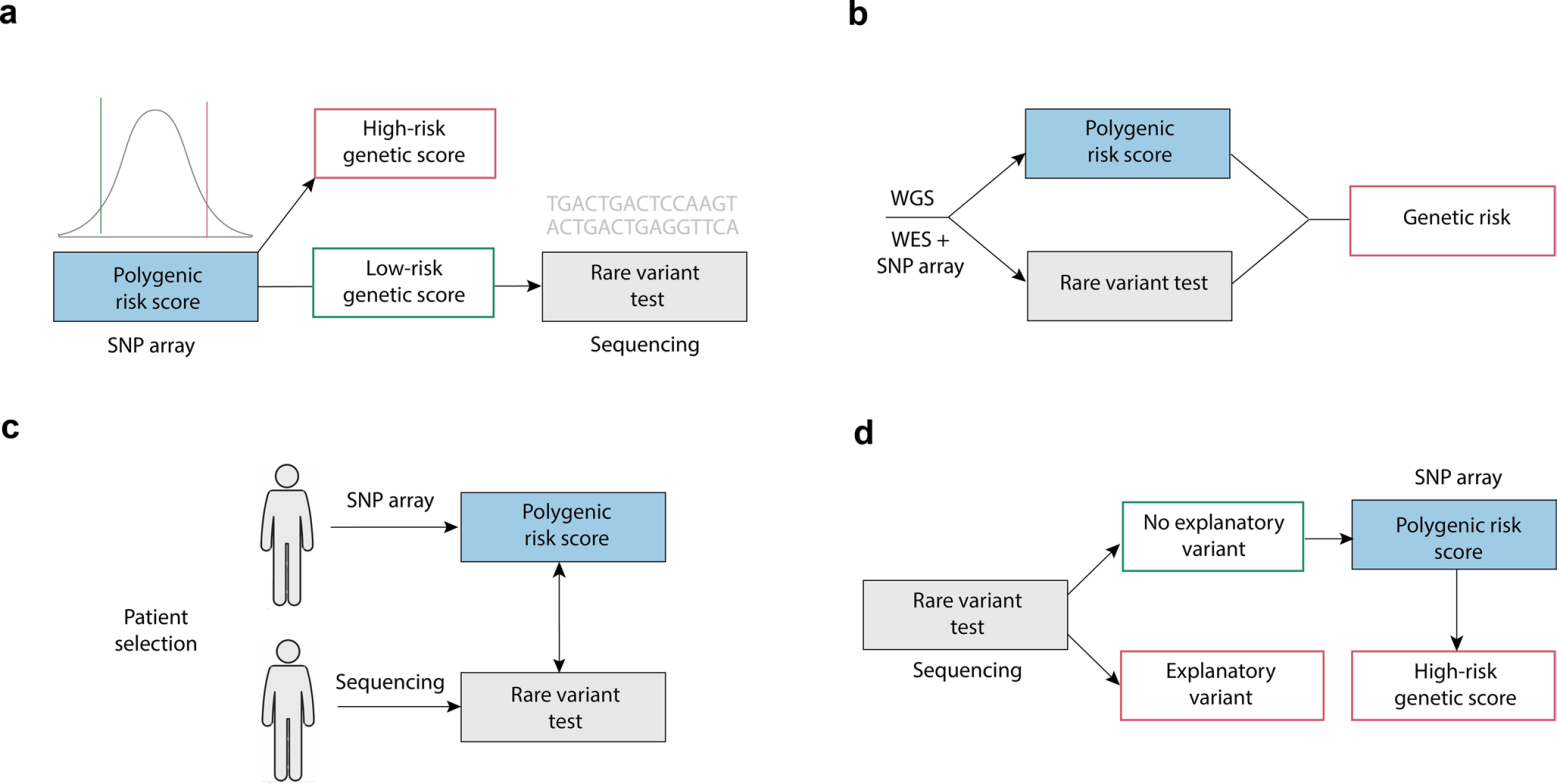
Potential scenarios of implementation of polygenic risk scores (PRS) within clinical genetic diagnostics. Scenarios are shown for (**a**) PRS as a prior test based on SNP array data; (**b**) PRS testing in parallel to traditional rare variant testing (e.g. in the form of standalone test or as part of whole-genome sequencing (WGS)); (**c**) either PRS or rare variant testing based on clinical presentation; (**d**) sequencing first approach with PRS in unexplained cases after rare variant testing

Several symptoms or disorders encountered in the clinic may have a monogenic component caused by a single high-penetrance variant, or a polygenic component, in which disease risk arises from the statistical contribution of many common variants (Abdellaoui et al. 2023), modified by environmental factors. Examples include developmental disorders, certain neurological disorders, autism, short stature and obesity, traits in which both rare and common variants play genetic variants contribute to disease risk. Analyses of several disorders, including neurodevelopmental disorders (Huang et al. 2024), hypercholesterolemia (Wu et al. 2021), type 2 diabetes and certain types of cancer (Lu et al. 2021), have shown that individuals carrying rare pathogenic variants tend to cluster in the low PRS range (i.e. those with a ‘favorable’ or low polygenic burden), whereas those with a high PRS are more likely to have symptoms consistent with a complex polygenic architecture rather than a single-gene cause. We observed similar associations in our genetic obesity clinic, where rare pathogenic variants in obesity genes were more than twice as common among in individuals in the low risk PRS segment (manuscript in revision). As such, PRS derived from SNP array genotyping may serve as an initial screening tool to help prioritize individuals who are more or less likely to benefit from subsequent rare variant testing through sequencing (Fig. 1a). Given the considerably shorter turnaround time and lower costs of SNP array genotyping ($55–70 per sample (Zhao et al. 2018) compared to whole-exome sequencing (WES, $600–700 per sample (Zhao et al. 2018), this may be a cost-effective approach to increase the diagnostic yield of WES. In this context, PRS does not provide a diagnostic result on its own, but can inform the likelihood that a patient’s phenotype has a predominantly polygenic rather than monogenic basis. Since PRS is a continuous probability score, a limitation of this approach is the often unclear optimal PRS threshold for defining ‘low’ and ‘high’ polygenic risk. Moreover, a high-risk PRS profile does not exclude the presence of a causal rare variant (Kang et al. 2024), therefore requiring a stringent PRS threshold to minimize the risk of missing such variants when sequencing is deferred in individuals with a high-risk PRS.

## Parallel with sequencing

While *scenario 1* suggests using PRS as an initial screen, an alternative approach is to integrate rare variant and common variant information in a single step. With lowering costs of whole-genome sequencing (WGS), this may become wider available as a first step in the diagnostic process (Lionel et al. 2018). A laboratory specialist can perform multiple tests (rare variant detection, CNV analysis, and common variant analysis) from a single assay, and data on rare genetic variants and PRS would be available simultaneously, reducing the need for separate assays like SNP arrays (Schobers et al. 2024) **(**Fig. 1b**)**. This enables the clinician to assess whether an explanatory rare pathogenic variant is present and, if not, whether the patient’s genetic background indicates an elevated polygenic risk. Moreover, while current PRS primarily rely on common variants (> 1% frequency), in principle a PRS could then also include (multiple) rare variants with stronger effects at once, blurring the distinction between a polygenic and monogenic etiology (Williams et al. 2024). Indeed, a PRS has shown to modify disease risk in monogenic disease carriers (e.g. BRCA (Kuchenbaecker et al. 2017) and MMR genes (Jenkins et al. 2021) for breast and colon cancer, respectively), showing the highest risk in rare variant carriers with a high-risk PRS (Kang et al. 2024). An important advantage of common and rare variant testing in parallel, is that a comprehensive genetic risk profile can be obtained, integrating information across the full spectrum of variant frequencies. Another strong advantage of this workflow (or of combining SNP array genotyping for PRS with WES for rare variant testing in parallel) is that all relevant analyses can be performed in a single step, shortening the diagnostic process. A potential downside, however, is the costs of (in some cases unnecessary) additional testing when no a priori distinction is made on whether a mono- or polygenic component is more likely. Also, WGS is an extensive assay that can identify many variants that are challenging and time-consuming to interpret, and a higher risk of secondary/unsolicited findings, putting a higher burden on laboratory specialists and patients. In addition, collecting and interpreting data on common variants in all patients through WGS leads to important bioinformatical challenges, such as data storage space and costs.

## Selective testing based on clinical characteristics

Alternatively, and likely the most realistic scenario at present, clinicians could select which test to prioritize (PRS vs. rare variant testing) and its corresponding analytic method (i.e. SNP array vs. sequencing) based on patient characteristics, rather than a one-size-fits-all workflow **(**Fig. 1c**)**. Based on criteria such as the presence of clear syndromic characteristics (e.g. dysmorphic features in combination with congenital anomalies or intellectual disability) or an early disease onset, rare variant testing via sequencing may be prioritized over PRS. Conversely, when these features are absent and a polygenic contribution is suspected, PRS testing derived from SNP genotyping may be carried out first, and sequencing could initially be omitted or considered a a secondary step. A clear advantage of this approach is that it supports a patient-centered and efficient diagnostic process tailored to individual pre-test likelihoods. This aligns well with current clinical practice, where diagnostic decisions are personalized, and specific tests are prioritized based on the characteristics of individual patient. A potential downside of this would be the need to maintain different workflows for various clinical indications instead of a single workflow (e.g. *scenario 2*). Also, this diagnostic workflow may take longer than when common and rare variant testing are performed simultaneously.

## PRS in unexplained cases

Lastly, and in contrast to *scenario 1*, rare variant testing via sequencing may be undertaken as a first test (‘sequencing-first’ approach), where SNP genotyping (or sequencing) of common variants for PRS analyses is only performed when no causal rare variant is found **(**Fig. 1d**)**. The advantage of prioritizing rare variant testing first is that it allows earlier diagnosis of conditions that typically present with a more severe phenotype and additional health risks. This also leads to a shorter timeframe for informing families about conditions with a Mendelian mode of inheritance, after which family counseling and segregation analysis can be initiated. Subsequent PRS testing after negative sequencing can then help identify patients in whom a polygenic contribution to disease risk is more likely. In clinical practice, patients with a negative result from rare variant testing may still undergo subsequent sequencing in the future when a genetic cause remains suspected, for instance due to a severe phenotype or strong family history. Such renewed testing occasionally identifies variants that were undetectable with earlier sequencing or in genes not yet associated with disease. For those with a high-risk PRS, however, repeated rare variant sequencing in the future may in this case be less likely to yield new findings. An important requirement of this approach would be a sufficiently powered PRS (based on a large GWAS), as noise in the PRS strongly limits its interpretation (Dudbridge 2013). A drawback of this approach is that costly rare variant sequencing is performed upfront, with a higher likelihood of unclear or unsolicited finding, which may be less clinically relevant when a polygenic component is more likely. In our department, we currently pilot PRS according to this scenario for obesity in those where no rare coding variant has been found.

## Discussion

Among these scenarios, the exact role of PRS testing will likely depend on the indication for genetic testing, particularly the extent to which both rare and common variants contribute to disease risk. Another disease-specific factor is the predictive accuracy of its relevant PRS, currently insufficient for many of these traits, although better-powered PRS are expected as the field advances. Importantly, this performance of PRS is worse when an individual is not of European ancestry (Martin et al. 2019), which may lead to unequal care in any of these scenarios, as non-European ancestries would receive a less accurate genetic test. Lastly, defining thresholds for what constitutes a high-risk PRS, when to interpret their disease risk as predominantly polygenic versus monogenic remains challenging, as does translating relative risks from PRS to absolute risks through accurate calibration (Uffelmann et al. 2025). Clear communication is essential to prevent misinterpretation of probabilistic information as deterministic, which could lead to unnecessary concern or clinical interventions. Ensuring that patients understand both the potential and the current limitations of PRS will be key for its responsible and ethically sound implementation. New large scale initiatives such as eMERGE (Lennon et al. 2024), GeNOVA studies (Vassy et al. 2023), Our Future Health (Ormondroyd et al. 2022) (as part of the UK government’s *Genome UK* initiative) and the All of US study (Investigators 2019), will help further establish risk thresholds and define the (clinical) role of PRS in different settings and populations. For meaningful clinical application, PRS should be viewed as one component within multifactorial risk assessment, ideally incorporate both genetic and clinical risk factors. This is exemplified by tools for breast cancer (CanRISK (Carver et al. 2021) and cardiovascular disease (Fuat et al. 2024), where clinical implementation is more advanced than for most other disorders. Their broader implementation should preferably be supported by randomized trials as has been done for primary care settings (Vassy et al. 2023), although this might not be feasible for all specific indications.

We note that discussions surrounding the role of PRS are not unique but parallel those occurring for other emerging genetic test. Similar discussions have taken place at our department with an upcoming role of epigenetic testing in diagnostics, which may also be done before, during or after DNA sequencing. Lessons learned from the implementation of previous diagnostic advances can inform and guide the integration of PRS into clinical practice. In our experience, this succeeds when clear discussion and collaboration takes place among professionals in both laboratory and clinic, ideally backed by (long-term) evidence and data on cost-effectiveness (yield/costs).

In conclusion, while PRS testing may not be ready for routine clinical diagnostics, clinicians should actively discuss and prepare for its potential integration, to ensure that these tools are used optimally in clinical practice, are grounded in evidence, and ultimately benefit the right patients.

## Data Availability

No datasets were generated or analysed during the current study.
